# Pediatric non-galenic pial arteriovenous fistula’s characteristics and outcomes: a systematic review

**DOI:** 10.1007/s00381-024-06352-5

**Published:** 2024-03-20

**Authors:** Garrett W. Thrash, Andrew T. Hale, Michael J. Feldman, Benjamin W. Saccomano, D. Jonah Barrett, Pedram D. Malenkia, Somnath Das, Georges Bouobda Tsemo, Jeffrey P. Blount, Brandon G. Rocque, Curtis J. Rozzelle, James M. Johnston, Jesse G. Jones

**Affiliations:** 1 Heersink School of Medicine, University of Alabama at Birmingham, Birmingham, AL USA; 2 Department of Neurosurgery, University of Alabama at Birmingham, FOT Suite 1060, 1720 2nd Ave S, Birmingham, AL 35294 USA; 3 Division of Pediatric Neurosurgery, Department of Neurosurgery, University of Alabama at Birmingham, Birmingham, AL USA; 4 Department of Diagnostic Radiology, University of Alabama at Birmingham, Birmingham, AL USA

**Keywords:** Pial arteriovenous fistula, Pial AV fistula, Non-galenic arteriovenous fistula, Non-galenic pial arteriovenous fistula, Pediatric cerebrovascular, Systematic review, Meta analysis

## Abstract

**Introduction:**

Pediatric non-galenic pial arteriovenous fistulas (pAVFs) are rare vascular malformations that are characterized by a pial arterial-venous connection without an intervening capillary bed. Outcomes and treatment strategies for pAVFs are highly individualized, owing to the rarity of the disease and lack of large-scale data guiding optimal treatment approaches.

**Methods:**

We performed a systematic review of pediatric patients (< 18 years at diagnosis) diagnosed with a pAVF by digital subtraction angiogram (DSA). The demographics, treatment modalities, and outcomes were documented for each patient and clinical outcome data was collected. Descriptive information stratified by outcome scores were classified as follows: 1 = excellent (no deficit and full premorbid activity), 2 = good (mild deficit and full premorbid activity), 3 = fair (moderate deficit and impaired activity), 4 = poor (severe deficit and dependent on others), 5 = death.

**Results:**

A total of 87 studies involving 231 patients were identified. Median age at diagnosis was 3 years (neonates to 18 years). There was slight male preponderance (55.4%), and 150 subjects (81.1%*) experienced excellent outcomes after treatment. Of the 189 patients treated using endovascular approaches, 80.3% experienced excellent outcomes and of the 15 patients surgically treated subjects 75% had an excellent outcome. The highest rate of excellent outcomes was achieved in patients treated with Onyx (95.2%) and other forms of EvOH (100%). High output heart failure and comorbid vascular lesions tended to result in worse outcomes, with only 54.2% and 68% of subjects experiencing an excellent outcome, respectively. *Outcomes were reported in only 185 patients.

**Conclusion:**

pAVFs are rare lesions, necessitating aggregation of patient data to inform natural history and optimal treatment strategies. This review summarizes the current literature on pAVF in children, where children presenting with heart failure as a result of high flow through the lesion were less likely to experience an excellent outcome. Prospective, large-scale studies would further characterize pediatric pAVFs and enable quantitative analysis of outcomes to inform best treatment practices.

**Supplementary Information:**

The online version contains supplementary material available at 10.1007/s00381-024-06352-5.

## Introduction

Pial arteriovenous fistulas (pAVF), also known as non-galenic arteriovenous fistulas, are vascular malformations that are distinct from arteriovenous malformations (AVM) due to the lack of a nidus between feeding artery and draining vein [1]. Unlike dural arteriovenous fistulas (dAVF), pAVFs involve parenchymal cerebral vasculature rather than meningeal [2]. pAVFs may drain through the vein of Galen (VOG) and still be differentiated from VOG malformations which are primarily fed by choroidal arteries. AV shunting by pAVFs predisposes to venous varix formation, which increases the risk of hemorrhage. pAVFs are rare lesions, contributing to only 1.6% of all brain vascular malformations [3, 4] Digital subtraction angiography (DSA) is recommended for diagnosis and characterization of the angio-architecture.

pAVFs are almost entirely congenital. The majority of diagnoses occur either shortly after birth due to heart failure or cerebral venous congestion/neurologic decline or following spontaneous intracranial hemorrhage later in life [5]. The molecular pathophysiology underlying pAVF formation *in utero* is not well characterized, but thought to involve perturbations in angiogenic growth factors and embryonic vascular morphogenesis [6–8]. The most frequently associated genetic mutations involve the hereditary hemorrhagic telangiectasia (HHT) genes (ENG, ACVRL and SMAD4) and *RASA1 --* which are also implicated in brain AVM and VOGM, respectively [9]. HHT is an autosomal dominant disease characterized by vascular malformations throughout the body [10]. *RASA1* variants affect the RAS/MAPK signaling pathway involved in vascular endothelial cell proliferation [11]. There is increasing recognition, overall, of the contribution of genetic factors to cerebrovascular disease more broadly.

The goal of pAVF treatment is disconnection of the shunt, either through open surgery or endovascular embolization. However, due to the rarity of the condition, there are no consensus guidelines for treatment. Surgical treatment of pAVFs is typically reserved for patients with intracranial hemorrhage causing mass effect and neurologic deterioration, but more commonly endovascular embolization is preferred [2]. In an effort to understand demographic, clinical, and radiological factors associated with treatment outcomes, we performed a systematic review of published pAVF cases in children.

## Methods

This systematic review followed PRISMA guidelines [12]. PubMed, CINAHL, Scopus, and Embase databases were queried without a date restriction. The protocol for the review was not registered. Search strategy included MeSH (Medical Subject Heading) terms related to pial arteriovenous fistulas and then translated across each database (see Appendix). Search results were then screened by title and abstract, then full text by two independent reviewers (G.T., J.B.) with discrepancies reviewed by a 3rd author (A.T.H). Articles were included based on the following criteria: (1) Available full text with English translation, (2) pediatric patients less than 18 years old, (3) individual patient data could be retrieved, (4) articles represented primary sources, and 4) pAVF was diagnosed by cerebral angiography. Only case reports, case studies, case series, and cohort studies were included in our analysis. Review articles, meta-analyses, non-human studies, conference papers, and abstracts without full text were excluded. Dependent variables were age, sex, race, cardiovascular disease, venous varix, cerebral hemorrhage, genetic disease, other vascular lesions, feeding artery name, draining vein location (deep/superficial), treatment modality, embolic agent, treatment success, number of stages, and procedural complications. Clinical outcome was identified in 187 patients (80.6%) and scored on a scale of 1–5 using the ranking system utilized by Hoh et al., to score pAVFs based on patient activity and deficit [5].

**Fig. 1:**
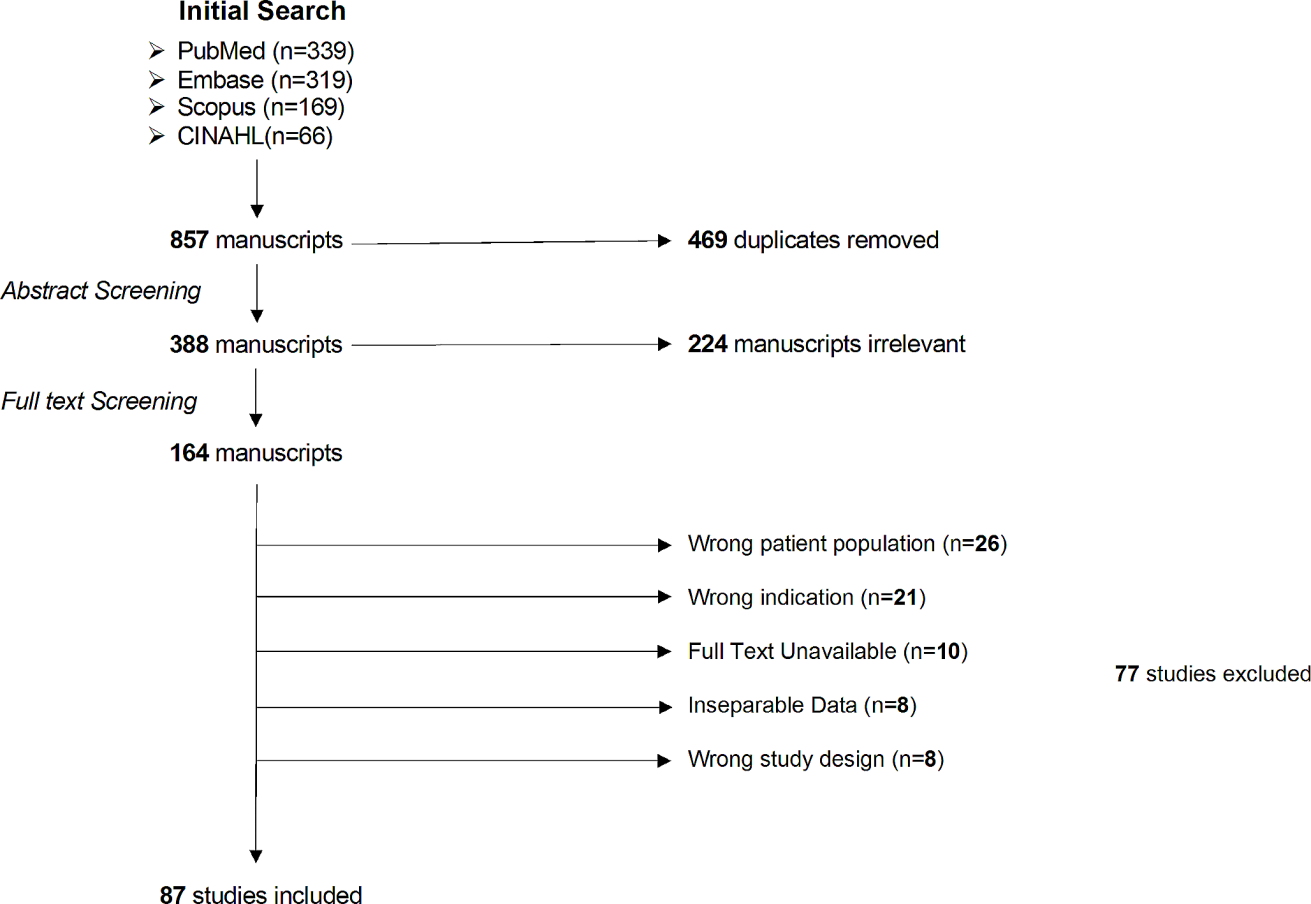
PRISMA flow diagram describing the results of our systematic review

## Results

There were 857 articles identified on initial screening (339 in PubMed, 319 in Embase,169 in Scopus, and 66 in CINAHL) and imported into Covidence. The 469 duplicates were automatically removed. Initial title and abstract screening removed another 224 articles, leaving 164 studies for full text review. Of these, 87 were included for data extraction based upon the aforementioned criteria. The other 77 manuscripts were removed due to the reasons found in Fig. 1. A summary of all included cases can be seen in supplemental material 1 [1–98].

In total, 231 pediatric patients harboring pAVF were identified. Median age was 3 years (< 1 month to 18 years). Slightly more patients were male (55.4%). Sixty-seven and one-half percent of subjects were symptomatic at diagnosis, most often from intracranial hemorrhage or high output heart failure (HOHF). Other presentations included headache (9.5%), seizures (12.1%), growth delay (5.2%) and macrocephaly (2.2%). All 39 patients presenting with HOHF were diagnosed in the neonatal period. Approximately half of subjects exhibited venous varices. One fifth of the cohort had an underlying genetic condition attributable to *RASA1* (12.6%) or HHT (5.6%) mutations, among others (Table 1). One patient was diagnosed with Moya-Moya syndrome and another with Encephalocraniocutaneous lipomatosis. The major feeding arteries to the pAVF included the middle cerebral (MCA) (16.9%), posterior cerebral (PCA) (11.7%), anterior cerebral (ACA) (10%) and posterior inferior cerebellar arteries (PICA) (5.2%). Another 28 patients (12.3%) had unrelated coexisting vascular lesions, such as AVM or intracranial aneurysm (Table 1).

**Table 1:** Patient demographics, genetic disease prevalence and angioarchitecture of the fistulae. Abbreviations include ACA- Anterior Cerebral Arter, CVD- cardiovascular disease, HHT- hereditary hemorrhagic telangiectasia, ICA- Internal Carotid Artery, MCA- Middle Cerebral Artery, N/A – not available, PCA- Posterior Cerebral Artery, PICA- Posterior Inferior Cerebellar Artery, SCA- Superior Cerebral Artery, SD-Standard Deviation, SSS- Superior Sagittal Sinus, and VOG- Vein of Galen

Variable	Value
**Total # of patients**	231
**Sex**	
Male	128 (55.4%)
Female	87(37.7%)
Missing	16(6.9%)
**Mean age (SD), years**	5.4(5.7)
**Median Age, years**	3.0
**Race**	Only 2 Cases Reported
**HOHF**	
Yes	39(16.9%)
No	192(83.1%)
**Venous Varix**	
Yes	114(49.3%)
No	117(50.7%)
**Hemorrhage**	
Yes	48(20.7%)
No	183(79.2%)
**All-Cause Mortality**	9 (3.9%)
**Genetic Disease**	
RASA1	29(12.6%)
HHT	13(5.6%)
Other	7(3.0%)
Not Mentioned	182(78.8%)
**Presentation**	
Asymptomatic	11(4.7%)
Symptomatic	156 (67.5%)
**Feeding Arteries**	
MCA	39(16.9%)
PCA	27(11.7%)
PICA	12(5.2%)
ACA	23(10.0%)
ICA	3(1.3%)
SCA	3(1.3%)
Other	29(12.6%)
Missing	96(41.6%)
**Venous Drainage**	
SSS	11(4.8%)
Transverse Sigmoid Sinus	5(2.2%)
VOG	11(4.8%)
Straight Sinus	12(5.2%)
Sylvian	4(1.7%)
Sigmoid Sinus	7(3.0%)
Other	30(13.0%)
Missing	151(65.4%)
**Coexisting Vascular Lesions**	
Yes	28(12.1%)
Diagnosed with Genetic Disease	12 (42.8%)

The median clinical follow up was 9 months, with a range of 3 months to 6 years. Of the 185 patients with reported outcomes data, 150 patients (81.1%) experienced an excellent outcome (Table 2). Among those with less than excellent outcomes, 8.6% were good outcome, 4.3% fair, 1.1% poor, and 4.9% experienced any-cause mortality. There were two intraoperative deaths. One from vessel perforation during endovascular treatment and the other from acute sinus thrombosis during open surgery [13, 14]. First time angiographic success (i.e., complete obliteration of the fistula) was achieved in 76.3% (135 patients). One patient experienced spontaneous involution. Twenty-nine subjects (12.6%) underwent a second treatment (Table 3) although whether staging was intentional could rarely be ascertained. Of those who underwent a 2nd treatment, 23 of those patients experienced complete obliteration. When accounting for obliterations after the 2nd treatment, the rate of complete obliteration rose to 89.2%. Procedural complications occurred in 21.6% of cases. Of the complications, 14.5% were transient (< 12 months duration) and 6.9% permanent. Major complications included cardiac arrest and venous sinus thrombosis. The infant with cardiac arrest was successfully resuscitated [15]. The patient who experienced venous thrombosis was treated with anti-coagulation and anti-platelet therapy and showed complete resolution at the 6-month follow-up [16]. Endovascular procedural complications consisted of distal embolic migration into the cerebral veins and lungs [17]. Subsequent venous congestion led to seizures in one subject [18]. Of the 13 permanent complications, 9 resulted in death. The 2 immediate peri-operatively mortalities were due to vessel perforation and acute sinus thrombosis. The 7 remaining patients died shortly post-op with 2 succumbing to brain death (not otherwise specified) and 5 to post-embolization hemorrhage. Six of the 9 patients were neonates.

**Table 2:** Treatment outcomes, obliteration rates, and complications during intervention. Outcome scores were adopted from Hoh, Putman, Budzik, et al., 2001 [5]. Outcomes were classified in the following manner: 1 = excellent (no deficit and full premorbid activity), 2 = good (mild deficit and full premorbid activity), 3 = fair(moderate deficit and impaired activity), 4 = poor (severe deficit and dependent on others), 5 = death. Those who received a 2nd treatment received obliteration

Variable	Value
Outcome Score	
1	150(81.1%)
2	16(8.6%)
3	8(4.3%)
4	2(1.1%)
5	9(4.8%)
Missing	46
**Angiographic Outcome**	
Obliteration	135(76.3%)
Spontaneous Angiographic Cure	1(0.5%)
Residual Pathology	12(6.7%)
Required 2nd Treatment	29(16.4%)
Missing	54
**Complications**	
None	146(78.4%)
Temporary	27(14.5%)
Permanent	13(6.9%)
Missing	48

**Table 3:** Treatment modalities and number of interventions needed for fistula obliteration. Abbreviations include: nBCA- n-Butyl cyanoacrylate and EvOH-ethyl vinyl alcohol

Variable	Value
Monomodal Therapy	
Embolization	189(81.8%)
Surgery	20(8.7%)
No Treatment	2(0.9%)
Radiosurgery	-
**Multimodal Therapy**	
Embolization + Surgery	10(4.3%)
Embolization + Radiosurgery	-
Embolization + Surgery + Radio Surgery	1(0.4%)
Total	11
**Number of Interventions**	
Single Intervention	195(84.4%)
2 or more interventions	31(13.4%)
**Type of Embolic Agent**	
Liquid	49(25.9%)
Coils	42(22.2%)
Liquid + Coils	40(21.2%)
Missing	58(30.7%)
**Type of Liquid Embolic**	
Onyx	21(22.8%)
Other Glue	4(4.3%)
nBCA	58(63.0%)
EvOH	9(9.8%)

Almost all subjects underwent treatment (Table 4). Endovascular embolization was the most common treatment, (81.8%) followed by open surgery (8.7%) and radiation (0.4%). Combination therapy was employed in 5.2% of cases. Among endovascular techniques, pure liquid (25.9%), coils (22.2%) and a combination of coils and liquid (21.2%) were used. Of the endovascularly treated patients, 80.3% of patients achieved an excellent outcome compared to 75% of surgically treated patients. Of the patients treated with multimodal therapy, 9 of the patients underwent the therapy as part of a second treatment option. Patients treated with Onyx, a type of liquid embolic system that consists of ethyl vinyl alcohol (EvOH), dimethyl sulfoxide (DMSO) and tantalum powder, experienced 95.2% excellent outcomes while all patients treated with other types of EvOH had excellent outcomes. HOHF reduced excellent outcomes to 45.8% and the presence of coexisting vascular lesions had only a 68.5% rate of excellent outcomes.

**Table 4:** Outcomes data of differing embolic treatments. The scores are represented by the following: 1 = excellent (no deficit and full premorbid activity), 2 = good (mild deficit and full premorbid activity), 3 = fair (moderate deficit and impaired activity), 4 = poor (severe deficit and dependent on others), 5 = death. Abbreviations include: nBCA- n-Butyl cyanoacrylate, EvOH-ethyl vinyl alcohol, and CVD- cardiovascular disease

Variable	Outcome = 1	Outcome = 2–5
**Surgery**	15(75%)	5(25%)
**Endovascular**	118(80.3%)	29(19.7%)
**Single Modality**	77(88.5%)	10(11.5%)
**Multiple Modalities**	9(81.8%)	-
**Coils**	49(77.8%)	14(22.2%)
**Liquid**	78(84.8%)	14(15.2%)
**nBCA**	46(79.3%)	12(20.7%)
**Onyx**	20(95.2%)	1(4.8%)
**Other EvOH**	7(100%)	-
**Presented with Venous Varix**	83 (82.2%)	18 (17.8%)
**Presented with Hemorrhage**	32 (80%)	8(20%)
**Presented with CVD**	11(45.8%)	13(54.2%)
**Reported Genetic Disease**	14(82.4%)	3(17.6%)
**Coexisting Vascular Lesions**	17 (68%)	8(32%)

## Discussion

Here we perform a systematic review of pAVF outcomes in children. While meta-analysis was not feasible owing to low sample size and the large number of case reports, we observed some patterns worth mentioning. First, those presenting with > 1 vascular malformation were likely to harbor an underlying genetic condition such as HHT or RASA1. Genetic testing should be considered in these cases [19]. Second, the presence of a venous varix was strongly associated with symptomatic and/or hemorrhagic presentation, implicating the varix as a high-risk feature.

Despite young average age at presentation, most patients (over 80%) experienced an excellent outcome. Patients treated with endovascular therapy alone or in combination achieved the highest rate of excellent outcomes, although several stages may be required to completely occlude the pAVF. Patients with HOHF were least likely to experience excellent outcome, possibly reflecting the severity of arteriovenous shunting and downstream effects on the brain and other organs. Poor outcomes clustered in the neonatal group, with most survivors demonstrating recovery from complications and normal development following complete treatment. Overall, this summative data represents the largest descriptive pediatric pAVF cohort.

The amount of missing data encountered in this study highlights the importance of standardized reporting that includes subject-level granularity. Common data elements reduce bias associated with missing results. This shortcoming is exaggerated in similar reviews of rare pathologies in the neurosurgical literature that by nature involve small sample size [6]. Another limitation is publication bias intrinsic to systematic reviews. Many potentially important variables could not be controlled for, such as length of follow-up, operator experience and treatment timing. Small sample size and the retrospective nature of all studies precluded statistical analysis.

## Conclusions

pAVFs are rare pediatric vascular anomalies with overall favorable outcome except when associated with heart failure or multifocal vascular lesions. Treatment appears well-tolerated and primarily involves endovascular embolization. This review encompasses the largest descriptive review of pediatric pAVFs. However, the included studies were entirely retrospective and primarily single center, limiting generalizability and with significant risk of bias. Further studies to delineate pAVF natural history and optimal treatment paradigm are needed and should conform to a standardized reporting format to facilitate metanalysis.

### Electronic supplementary material

Below is the link to the electronic supplementary material.

Supplementary Material 1

## Data Availability

Datasets and search terms for this systematic review can be found in the appendix below.
