# Supplementary material for: Pediatric non-galenic pial arteriovenous fistula’s characteristics and outcomes: a systematic review
Source: Childs Nerv Syst. 2024 Mar 20;40(6):1721–9. doi: 10.1007/s00381-024-06352-5 (PMC11111522; doi:10.1007/s00381-024-06352-5)
Supplement: Supplementary file 1 — Supplementary Material 1 [file 381_2024_6352_MOESM1_ESM.docx]

**Appendix:**

**Table 1.** Summary of including studies including their study design, year of publication, and number of patients included from the number of total cases in each study.

| **Study ID** | **Number of total cases** | **Number of cases included** | **Study Design** |
| --- | --- | --- | --- |
| Ago 2017^20^ | 1 | 1 | Case Report |
| Aguilar 2011^21^ | 1 | 1 | Case Report |
| Akamatsu 2018^22^ | 1 | 1 | Case Report |
| Altschul 2014^23^ | 11 | 2 | Case Series |
| Alurkar 2016^24^ | 3 | 3 | Case Series |
| Auyeung 2003^25^ | 1 | 1 | Case Report |
| Bankole 2021^26^ | 1 | 1 | Case Report |
| Batista 2002^27^ | 1 | 1 | Case Report |
| Berestov 2022^28^ | 1 | 1 | Case Report |
| Bongetta 2015^29^ | 1 | 1 | Case Report |
| Cooke 2012^30^ | 1 | 1 | Case Report |
| Coubes 1996^31^ | 1 | 1 | Case Report |
| Cuoco 2021^32^ | 1 | 1 | Case Report |
| daSilvaMartins 2015^33^ | 2 | 1 | Case Report |
| Demartini 2021^34^ | 1 | 1 | Case Report |
| Deniwar 2022^35^ | 15 | 4 | Case Series |
| Feng 2016^36^ | 2 | 2 | Case Series |
| Fry 2023^37^ | 1 | 1 | Case Report |
| Garcia-Monaco1995^38^ | 4 | 4 | Case Series |
| Garel 2005^39^ | 3 | 3 | Case Series |
| Ghorbani 2019^40^ | 1 | 1 | Case Report |
| Goel 2018^41^ | 14 | 8 | Case Series |
| Gonzalez 2013^42^ | 2 | 1 | Case Report |
| Guerra 2011^43^ | 1 | 1 | Case Report |
| Guimaraens 2011^44^ |  | 1 | Case Report |
| Han 2013^45^ | 1 | 1 | Case Report |
| Hatayama 2018^46^ | 1 | 1 | Case Report |
| Ito 2000^47^ | 1 | 1 | Case Report |
| Izzo 2006^48^ | 1 | 1 | Case Report |
| Jin 2021^49^ | 20 | 13 | Case Series |
| Koroglu 2006^50^ | 1 | 1 | Case Report |
| Kalra 2020^51^ | 1 | 1 | Case Report |
| Keskin 2015^52^ | 1 | 1 | Case Report |
| Komiyama 2016^13^ | 15 | 3 | Case Series |
| Kraneburg 2014^53^ | 1 | 1 | Case Report |
| Kuwabara 2020^54^ | 1 | 1 | Case Report |
| Lee 2008^55^ | 2 | 1 | Case Report |
| Lee 2012^56^ | 1 | 1 | Case Report |
| Li 2018^57^ | 1 | 1 | Case Report |
| Li 2022^58^ | 1 | 1 | Case Report |
| Limaye 2004^59^ | 5 | 3 | Case Series |
| LoPresti 2015^60^ | 1 | 1 | Case Report |
| Lv 2010^61^ | 16 | 10 | Case Series |
| Lylyk 2017^62^ | 2 | 1 | Case Series |
| Madsen 2013^63^ | 5 | 5 | Case Series |
| Maejima 2018^64^ | 1 | 1 | Case Report |
| Mahmoud 2018^65^ | 2 | 2 | Case Series |
| Miyamoto 2023^66^ | 1 | 1 | Case Report |
| Morales-Gomez 2017^67^ | 1 | 1 | Case Report |
| Naik 2015^68^ | 1 | 1 | Case Report |
| Nakiri 2010^69^ | 1 | 1 | Case Report |
| Nesbit 1998^70^ | 12 | 2 | Case Series |
| Newman 2011^71^ | 2 | 2 | Case Series |
| Okazaki 2019^72^ | 1 | 1 | Case Report |
| Paramasivam 2013^73^ | 16 | 16 | Case Series |
| Pedicelli 2017^74^ | 1 | 1 | Case Report |
| Phelps 2021^75^ | 1 | 1 | Case Report |
| Pillai 2006^15^ | 1 | 1 | Case Report |
| Puccinelli 2019^76^ | 1 | 1 | Case Report |
| Requejo 2015^77^ | 10 | 10 | Case Series |
| Requejo 2023^78^ | 9 | 9 | Case Series |
| Ryu 2021^79^ | 1 | 1 | Case Report |
| Sabrina 2023^80^ | 1 | 1 | Case Report |
| Sailou 2017^81^ | 43 | 42 | Case Series |
| Sarigecili 2019^82^ | 1 | 1 | Case Report |
| Sato 2021^83^ | 1 | 1 | Case Report |
| Selvamurugan 2021^84^ | 1 | 1 | Case Report |
| Smith 2013^85^ | 1 | 1 | Case Report |
| Sugimoto 2015^86^ | 1 | 1 | Case Report |
| Tabatabai 2008^87^ | 1 | 1 | Case Report |
| Tomycz 2012^88^ | 1 | 1 | Case Report |
| Tripathy 2015^89^ | 1 | 1 | Case Report |
| Venkatesulu 2019^90^ | 1 | 1 | Case Report |
| Vinuela 1987^17^ | 8 | 4 | Case Series |
| Walcott 2011^91^ | 7 | 7 | Case Series |
| Wang 2004^92^ | 3 | 2 | Case Series |
| Yan 2021^93^ | 1 | 1 | Case Report |
| Yang 2013^16^ | 1 | 1 | Case Report |
| Ye 2018^94^ | 8 | 3 | Case Series |
| Yokota 2009^95^ | 1 | 1 | Case Report |
| Youn 2010^18^ | 11 | 5 | Case Series |
| Zaidi 2015^14^ | 6 | 6 | Case Series |
| Zenteno 2018^96^ | 10 | 1 | Case Series |
| Zhang 2013^97^ | 1 | 1 | Case Report |
| Zuccaro 2010^98^ | 2 | 2 | Case Series |

**Search Criteria**

**Pubmed:**

"Arteriovenous Fistula"[Mesh] OR anastomosis-arteriovenosa[tiab] OR arterial-venous-anastomosis[tiab] OR arterio-venous-anastomosis[tiab] OR arterio-venous-aneurysm[tiab] OR arterio-venous-fistula[tiab] OR arterio-venous-fistulae[tiab] OR arterio-venous-fistulas[tiab] OR arteriovenous-anastomosis[tiab] OR arteriovenous-aneurysm[tiab] OR arteriovenous-crossing[tiab] OR arteriovenous-fistulae[tiab] OR arteriovenous-fistulas[tiab] OR artery-vein-fistula[tiab] OR av-anastomosis[tiab] OR av-aneurysm[tiab] OR AV-fistula[tiab] OR AV-fistulae[tiab] OR “AV fistula”[Title/Abstract:~3] OR “AV fistulas”[Title/Abstract:~3] OR “Arteriovenous fistula” [Title/Abstract:~3] OR AVF[tiab] OR pAVF[tiab] AND ("Clinical Trial" [PT:NoExp] OR "clinical trial, phase i"[pt] OR "clinical trial, phase ii"[pt] OR "clinical trial, phase iii"[pt] OR "clinical trial, phase iv"[pt] OR "controlled clinical trial"[pt] OR "multicenter study"[pt] OR "randomized controlled trial"[pt] OR "Clinical Trials as Topic"[mesh:noexp] OR "clinical trials, phase i as topic"[MeSH Terms:noexp] OR "clinical trials, phase ii as topic"[MeSH Terms:noexp] OR "clinical trials, phase iii as topic"[MeSH Terms:noexp] OR "clinical trials, phase iv as topic"[MeSH Terms:noexp] OR "controlled clinical trials as topic"[MeSH Terms:noexp] OR "randomized controlled trials as topic"[MeSH Terms:noexp] OR "early termination of clinical trials"[MeSH Terms:noexp] OR "multicenter studies as topic"[MeSH Terms:noexp] OR "Double-Blind Method"[Mesh] OR ((randomised[TIAB] OR randomized[TIAB]) AND (trial[TIAB] OR trials[tiab])) OR ((single[TIAB] OR double[TIAB] OR doubled[TIAB] OR triple[TIAB] OR tripled[TIAB] OR treble[TIAB] OR treble[TIAB]) AND (blind*[TIAB] OR mask*[TIAB])) OR ("4 arm"[tiab] OR "four arm"[tiab])) OR ("Case Reports" [Publication Type] OR case-report*[Title/Abstract] OR case-series[Title/Abstract] OR case-study[Title/Abstract] OR case-studies[Title/Abstract])

**Embase:**

('arteriovenous fistula'/exp OR (AVF OR pAVF OR ((arteriovenosa OR arterial-venous OR arterio-venous OR arteriovenous OR artery-vein OR AV) NEAR/3 (anastomosis OR fistula* OR aneurysm OR crossing))):ti,ab) AND (pial OR non-galenic):ti,ab

**Scopus:**

TITLE-ABS-KEY(AVF OR pAVF) OR TITLE-ABS-KEY((arteriovenosa OR arterial-venous OR arterio-venous OR arteriovenous OR artery-vein OR AV) NEAR/3 (anastomosis OR fistula* OR aneurysm OR crossing))

TITLE-ABS-KEY(pial OR non-galenic)

**CINAHL**

(MH "Arteriovenous Fistula") OR (AVF OR pAVF) OR ((arteriovenosa OR arterial-venous OR arterio-venous OR arteriovenous OR artery-vein OR AV) N3 (anastomosis OR fistula* OR aneurysm OR crossing))):ti,ab) AND pial OR non-galenic
